# Effect of student-led health interventions on patient outcomes for those with cardiovascular disease or cardiovascular disease risk factors: a systematic review

**DOI:** 10.1186/s12872-020-01602-1

**Published:** 2020-07-11

**Authors:** Jenni Suen, Stacie Attrill, Jolene M. Thomas, Matilda Smale, Christopher L. Delaney, Michelle D. Miller

**Affiliations:** 1 Nutrition and Dietetics, College of Nursing and Health Sciences, Flinders University, GPO Box 2100, Adelaide, SA 5001 Australia; 2 Speech Pathology, College of Nursing and Health Sciences, Flinders University, GPO Box 2100, Adelaide, SA 5001 Australia; 3 Vascular Surgery Unit, Flinders Medical Centre, Flinders Drive, Bedford Park, South Australia 5042 Australia

**Keywords:** Student clinic, Cardiovascular risk factor, Patient outcomes, Clinical outcomes

## Abstract

**Background:**

As the need for health care services rise, alternative service delivery models such as student-led health interventions become attractive alternatives to alleviate the burden on healthcare. Predominantly, student-led health interventions were free clinics servicing socially disadvantaged communities in the USA. A 2015 systematic review identified that students value these student-run clinics and reported skill and knowledge attainment from participating. Previous research has reported on patient satisfaction outcomes, but less frequently about the clinical outcomes patients accrue from these student-delivered services. As cardiovascular disease is the leading cause of death worldwide, this review aimed to explore the effectiveness of student-led health interventions through examining their impact on objective clinical outcomes, using the case of patients at risk of, or with, cardiovascular disease.

**Methods:**

A systematic literature search was conducted in eight electronic databases to identify student-led health interventions conducted on adults with a cardiovascular disease risk factor or established cardiovascular disease, and a clinical outcome of interest. Through double-blinded screening and data extraction, sixteen studies were identified for synthesis.

**Results:**

The majority of student-led health interventions for patients at risk of cardiovascular disease demonstrated a positive impact on patient health. Statistically significant changes amongst patients at risk of cardiovascular disease appeared to be associated with student-led individualised intervention or group-based interventions amongst patients with diabetes or those who are overweight or obese. The evidence was of moderate quality, as included studies lacked a control group for comparison and detail to enable the intervention to be replicated.

**Conclusions:**

Future research applying a student-led health intervention through a randomised control trial, with rigorous reporting of both student and patient interventions and outcomes, are required to further understand the effectiveness of this alternative service delivery model.

## Background

Student-led health interventions, which provide health-practitioner students with opportunities to learn professional skills and competencies through delivering services, are commonly applied with a dual purpose, to also fulfil a service delivery gap in the healthcare system [1, 2]. A national survey in 2005 revealed that amongst medical schools in 25 states of the USA, over 100 student-run free clinics run by student volunteers existed, that provided healthcare services to the underinsured or socially disadvantaged populations [1, 3].

Amongst systematic review and research article literature, student-led health interventions are widely reported to benefit student learning through improving communication skills [4], knowledge [4, 5], confidence [6] and professional identity [6]. The robustness of this evidence can be appraised according to the Kirkpatrick model of evaluating training, which suggests four levels of evaluation of training [7]. The first level assesses the participant’s reaction, the second level assesses learned skills, the third assesses how learning is applied as a behaviour and the highest level assesses the wider benefit of the training, for example on the organisation or career trajectory [7].

Patient satisfaction questionnaires are the most common primary outcome measure used to assess effectiveness of student-led services for patients. This form of evaluation however can only capture the participant’s reaction to the service and does not capture higher level evaluation, including the ability of the intervention to facilitate learning and behaviour change [8]. Accordingly, the results from patient satisfaction questionnaires, often suggest that overall patients are satisfied with student services [9–14] providing the lowest level of evaluation according to the Kirkpatrick model [8]. Satisfaction may also be influenced by personal factors such as the patient’s ability to access healthcare [15] or communication and interpersonal skills of the students. These factors may have greater influence on patient satisfaction than the patient’s clinical outcome [16]. Therefore, measures of patient satisfaction alone are unable to inform whether student-led healthcare provide specific clinical benefits to patients who are recipients of these services. Understanding whether clinical markers of disease can be improved through student-led health interventions can help researchers to understand the true effectiveness of this style of health intervention for patients, which is a commonly applied service provision model to address healthcare service gaps or for socially disadvantaged communities [1, 3].

Given that the cost of healthcare is predicted to rise in many countries due to the increasing chronicity of health conditions and the aging population, this knowledge would assist in determining if this alternate service delivery model is a feasible substitute to usual healthcare provision. Cardiovascular disease as the leading cause of death amongst adults worldwide [17], along with its associated risk factors such as hypertension, hyperlipidaemia and diabetes, serve as a fitting disease population to demonstrate whether student-led services can impact patient outcomes and thus potentially elevate this burden on healthcare.

This review aimed to investigate the effectiveness of student-led health interventions through examining their impact on objective clinical outcomes of patients at risk of, or with, cardiovascular disease.

## Methods

### Selection criteria

The study protocol was registered on PROSPERO (ID: CRD42019115327). This review included: English language studies, available in full text, conducted on adult participants (aged 18 years or older) with a diagnosed cardiovascular disease risk factor (i.e. diabetes, hypertension, hyperlipidaemia, overweight or obesity) or established cardiovascular disease (i.e. coronary heart disease, stroke, heart failure) where objective patient outcomes where reported (i.e. blood pressure, lipid studies, Haemoglobin A1c, body weight, body mass index, readmission rate). As this review aimed to explore the effectiveness on objective patient outcomes, studies that exclusively reported student outcomes or subjective patient outcomes such as patient satisfaction and perception only, were excluded. This study includes student interventions that involve student participants, regardless of the level of supervision provided or the level of patient interaction provided to the student participant. Therefore, students providing patient care in both supervised and unsupervised settings have been included. Student clinics that involve students responsible for collection of information up to the point where the intervention is provided also have been included. All these forms of student participation have been termed as student clinics throughout the literature, and therefore have been included in this review.

### Search strategy

To identify studies for inclusion, a systematic literature search was conducted in the following electronic databases; Medline, Scopus, ProQuest Health & Medicine, Informit Health Database Collection, CINAHL, PsycINFO, Cochrane Library and Web of Science. Key search terms, informed by previous reviews [18, 19], were a combination (using OR) of synonyms for “*student-led intervention*” such as “*directed*” combined with (using AND) the Medical Subject Heading (MeSH) including related MeSH headings such as “*diabetes mellitus*” and “*cardiovascular disease*” combined (using OR). No date limit was applied to the search and thus included studies published up to 19th August 2019.

An example of a search strategy applied to Cochrane Library: ((student* near/4 (led or run or managed or facilitated or directed)) and (clinic or clinics or service* or consult* or care or healthcare or program* or practice* or model* or initiative* or intervention* or promotion* or centre* or center*)) and obes* or “morbid* obes*” or overweight or diabet* or hypertensi* or “blood pressure” or heart or cardiac or cardiovascular or coronary or vascular or stroke or “cerebrovascular accident*” or arrhythmi* or atrial or myocardial or hyperlipidemia or cholesterol or Hypercholesterolemia or hypertriglyceridemia*). See ‘Search Strategies’ in Additional file 1 for search strategies applied to each database.

### Screening and data extraction

Identified studies were imported to Covidence [20] for screening and data extraction by two teams of two to three blinded reviewers and screened according to the inclusion and exclusion criteria detailed above. Reference lists of included papers were screened to identify other potentially relevant publications for inclusion. Relevant publications were imported into Covidence [20] for double-blinded screening and data extraction.

For included studies, each team of blinded reviewers extracted data to gather information about the study design, the methods, the population, baseline characteristics and outcomes, and documented the data on Covidence [20]. Data collected on the intervention included the patient care provided as well as any student learning, orientation, training or supervision provided to students to enable them to deliver the patient care. See ‘Data Extraction Protocol’ in Additional file 1. The consensus between the data extracted from both teams was also completed in Covidence [20]. The consensus data extracted was used to create the tables and form the synthesis.

### Study quality assessment

To assess study quality of included studies, two independent reviewers critiqued each study using The Joanna Briggs Institute Critical Appraisal Tools [21] according to the study design. The tools include checklists that consist of key questions to identify the possibility of bias based on the design of the study that may impact on the interpretation of the results [21]. Each question on the checklist was allocated, ‘Yes’, ‘No’, ‘Unclear’ or ‘Not applicable’. Each reviewer recorded 1) the answer to each question on the checklist, 2) evidence from the paper that supported their answer, 3) page number that the evidence could be found on, 4) other comments, on a standard Microsoft Excel spread sheet prepared for both reviewers prior to independent critique. Reviewers allocated ‘Yes’ when studies met the criteria, ‘No’ when studies did not meet the criteria or did not report on the criteria or ‘Unclear’ when the studies mentioned criteria but did not provide all the detail required to eliminate bias from the criteria.

Each study appraised was discussed and any discrepancies were resolved with a third reviewer. The study quality was considered in the discussion to appropriately inform limitations of the evidence available and the level of evidence according to the GRADE method of reporting the quality of evidence [22]. Evidence can be categorised into one of four grades of evidence according to the GRADE method; high, moderate, low and very low; in the confidence that the researchers have on the estimated effect [22].

### Synthesis and interpretation

Due to the heterogeneity in the student-led health interventions, study design, outcomes of significance and data available, a meta-analysis was not suitable. Therefore, a narrative synthesis of the evidence gathered is presented.

The magnitude of change in clinical outcome considered to be clinical significant was guided by current literature. Specifically a 0.5% reduction in HbA1c is recognised as a clinically significant change by both the American Diabetes Association and National Institute for Health and Clinical Excellence [23]. Additionally through a randomised controlled trial, a 0.5% reduction in HbA1c was associated with a reduced risk in composite all-cause mortality, stroke and non-fatal myocardial infarction (HR 0.84, *p* = 0.027) [24].

With respect to blood pressure, a meta-regression analysis suggested that each 5 mmHg reduction in SBP and 2 mmHg reduction in DBP was respectively associated with a 13 and 12% less risk of composite cardiovascular endpoint [25].

Lipid analysis shows that each 38.7 mg/dL reduction in LDL is associated with a 23% lower risk of major vascular events [26]., while from an evidence-based review, 5% weight loss was related to improvements in blood pressure as well as HDL and LDL [27]. In addition, a waist circumference reduction of greater or equal to 3 cm demonstrated improvements in metabolic syndrome [28, 29], lowering cardiovascular risk.

Therefore, a 0.5% reduction in HbA1c, 5% weight loss, ≥3 cm reduction in waist circumference, change in LDL of ≥38.7 mg/dl and ≥ 5 mmHg reduction in systolic blood pressure and ≥ 2 mmHg in diastolic blood pressure were deemed clinically meaningful for the purposes of this review.

## Results

### Study selection

**Fig. 1:**
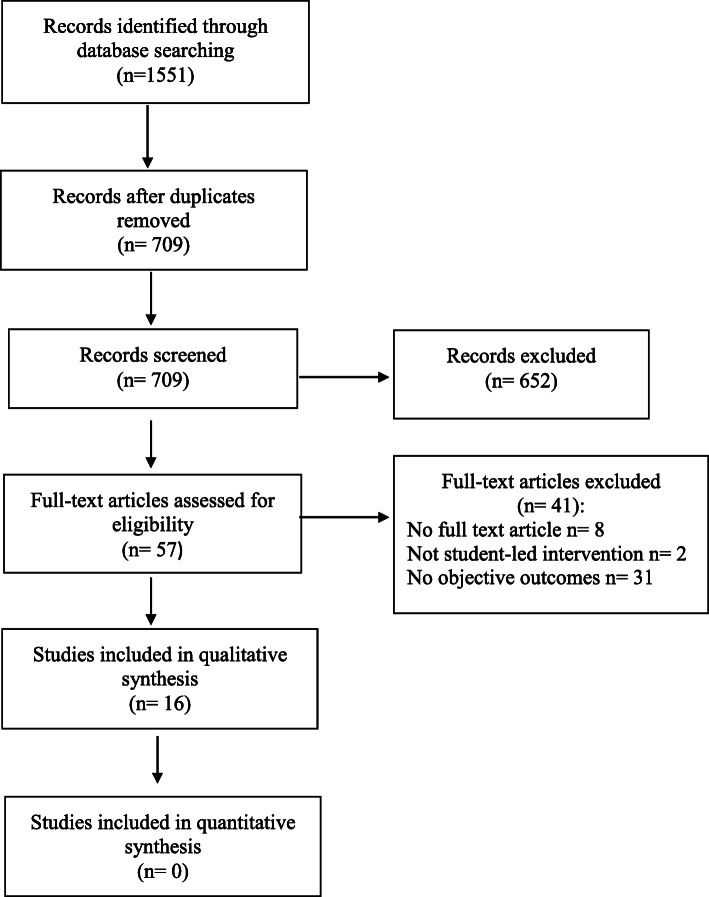
PRISMA Flow Diagram of Search Strategy

The search yielded 709 records for screening after duplicate removal (Fig. 1). Following full text screening and handsearching of reference lists of included studies, 16 studies were deemed eligible for qualitative synthesis (Fig. 1). Common patient outcomes measured across 14 studies included Haemoglobin A1c, total cholesterol, low-density lipoprotein, high-density lipoprotein, blood pressure, body weight and body mass index (BMI). Only one study reported on hospital readmission rate amongst participating patients as the primary outcome [30].

### Study characteristics

The 16 included studies captured 2504 patients with a cardiovascular disease or cardiovascular risk factor and 656 students that participated in a student-led health intervention, amongst studies that reported the number of participants. The number of student participants was not reported amongst all included studies.

As the included studies addressed cardiovascular risk factors, 11 studies focused on patients with diabetes, two focused on patients who were overweight, or obese and three single studies included patients with hyperlipidaemia, hypertension or heart failure.

Student-led health interventions were primarily located in a clinic setting [31–44]. Of the student-led clinics, five studies described the service provided as a “free clinic” [33, 36, 41, 42, 44]. The exceptions to the clinic-based student-led services included one study that provided student-led home visits [45] and one study that provided a student-led discharge consultation [30]. All student-led health interventions described were located in the USA, except Adam et al. [31] which was located in the United Kingdom.

Student-led interventions consisted of medication reviews [31, 38, 40], counselling through an interprofessional team of students [35, 38], medical appointments [36, 43, 44], home visitations for medication and dietary review [45] or a program of medical care and group education classes [39] or an individual program of phone calls and group classes [37].

Student involvement, training provided prior and level of supervision provided during the intervention varied amongst the student-led interventions. In some studies, students were provided a training program before taking upon the role of delivering the intervention [32, 37, 40, 45]. Other students were supervised by a health professional [31, 39] or mentored by a senior peer [33, 34] whilst providing the intervention. In two studies, students were mainly involved in collecting information prior to an intervention administered by health professionals [36, 38]. Student involvement, training and supervision were not clearly reported amongst six studies [30, 35, 41–44]. A ‘Results Summary Table’ can be found in Additional file 1.

### HbA1c

Amongst patients with diabetes, a statistically significant mean reduction in HbA1c that ranged from − 0.9 to − 1.7% (*p* = 0.008 to < 0.001) was observed when individual counselling [34, 40] or medical appointments [43, 44] or multiple pharmacy appointments [40] were provided as student-led interventions (Table 1). First and second year medical students provided individualised disease and nutrition counselling to diabetic patients in a student-run free clinic in an urban community health centre [34]. Students continued care outside the clinic via phone call contact, answering patient’s questions [34]. Student pharmacists who were undergoing a six-week placement in the community in rural Colorado provided individualised diabetes self-management sessions to diabetes patients [40]. Overall, patients were provided with six sessions over a six-month period [40]. Medical students provided medical appointments through a student clinic [43, 44]. Students involved in Mehta and colleagues [44] provided two or more appointments, whilst the number and frequency of medical appointments provided to patients was not described by Smith et al. [43].

**Table 1:** Changes in HbA1c with intervention observed across studies

Author, year	HbA1c (%)				
	Baseline (Mean ± SD)	N	Outcome (Mean ± SD)	N	*P*-value
Adams et al., 2015 [31]	7.3 ± 3.2	67	7.3 ± 3.2	67	NP
Gorrindo et al., 2014 [34]	9.6 ± 2.3	45	7.9 ± 1.8	45	< 0.0001
Janson et al., 2009 [35]	7.7 ± 1.7	384	7.7 ± 1.6	384	NP
Kahkoska et al., 2018 [36]	9.7 ± 1.6	29	9.2 ± 1.4	29	NP
Lee et al., 2016 [37]	9.2 ± NP	22	8.0 ± NP	22	NP
Martin et al., 2016 – uncontrolled [38]	9.3 ± NP	NP	7.6 ± NP	NP	NS
Martin et al., 2016 – controlled [38]	6.2 ± NP	NP	6.4 ± NP	NP	0.004
Mehta et al., 2016 [44]	9.5 ± 2.3	21	8.3 ± 2.2	21	0.008
Nuffer et al., 2012 [40]	7.7 ± 2.0	346	6.8 ± 1.1	346	< 0.001
Nagelkerk et al., 2018 [39]	7.3 ± NP	250	7.2 ± NP	250	0.346
Smith et al., 2014 [43]	9.2 ± 2.5	157	8.2 ± 2.2	157	< 0.001
Stroup et al., 2003 [45]	11.2 ± 1.3	30	10.0 ± 2.0	30	NP

*NP* Not provided, *NS* Not significant

Two studies reported a mean reduction in HbA1c of − 0.5%, *n* = 8 [36] and − 1.1%, *n* = 18 [38] but did not report statistical significance represented as *p*-values. Kahkoska and colleagues [36] provided a student-run free clinic which included a triaged medical appointment by a physician assistant, nursing students, a pharmacy resident and medical student. The student intervention was followed by a shared medical appointment prior to seeing the attending endocrinologist [36]. Medical appointments were shared amongst cohorts of four to 12 patients with diabetes where students led a discussion on diabetes self-management [36]. Martin and colleagues [38] provided multidisciplinary counselling (i.e. physician, pharmacist, nurse and dietitian) followed by student pharmacist review. The pharmacy student provided follow up the next day making recommendations and answering any patient questions [38].

Three studies found non-statistically significant changes in HbA1c when the student-led intervention was provided [31, 35, 39, 45]. When pairs of pharmacy students provided level two and three medication reviews to 67 patients with diabetes the intervention group had a lower post-intervention HbA1c (56.32 ± 11.5%) than the control group (59.68 ± 13.2%, *n* = 66, *p* = 0.14) who received usual care [31]. Each student pair attended to four patients [31]. Within intervention group change was − 0.49% compared to − 0.03% in the control group [31]. Pharmacy students who provided home visits or a telephone call to check patient compliance to medications and diet as instructed by a dietitian led to a mean − 1.2% reduction in HbA1c amongst 30 patients [45]. A similar mean reduction of − 0.8% in HbA1c was achieved when patients did not receive this compliance check from pharmacy students [45]. The frequency of the intervention over the 2-year intervention period was not reported [45].

Medical, pharmacy and physician assistant students who were on a 4–8 week placement depending on their discipline were involved in leading a 12-month group program for 221 patients with diabetes, supervised by a dietitian, led to no change in HbA1c (7.3 to 7.2%) [39]. Additionally students provided individualised care through phone calls supervised by nursing staff [39]. Details of the group program or the phone calls and the frequency and duration of the contact were not detailed [39].

Ten of 11 studies demonstrated a clinically significant change in HbA1c (− 0.5%) when a student-led health intervention was applied [23].

### Lipid studies

When lipid biochemistry was additionally measured amongst patients with diabetes after six individual pharmacy student-led appointments over 6 months [40] or medical appointments by medical students [43], statistically significant (*p* < 0.001) mean reductions were observed in total cholesterol of − 14.1 mg/dl [40], LDL of -12 mg/dl and − 28.2 mg/dl, TG − 31.2 mg/dl and 70.6 mg/dl were achieved respectively were achieved. The type of care provided at these appointments was not detailed.

A free medical student-led intervention providing patients with education, specialist consultations and dispensing of free medications led to a statistically significant mean reduction in LDL of − 34.5 mg/dL from 135.8 ± 37.2 mg/dl to 101.3 ± 34.6 mg/dl (*p* < 0.001) amongst 96 patients with hyperlipidaemia [41]. Additionally, a statistically and clinically significant (< 100 mg/dL) [46], mean LDL reduction of − 38.9 mg/dL from 133.6 mg/dl to 94.7 mg/dL, (*P* < 0.001) was observed in 72 participants who also had diabetes [41].

### Body weight outcomes

Two studies observed an increase in BMI of 0.5 kg/m^2^ (*n* = 19, *p* = 0.04, 44) and 0.3 kg/m^2^ (*n* = 238, *p* = 0.025) [39] when less than two medical appointments [44] or a 12 month program of medical care and group education [39] were provided respectively by medical students for patients with diabetes. Baseline and outcome weight, an important co-marker of health alongside BMI amongst patients with diabetes, were not reported amongst these two studies.

Amongst overweight or obese patients, Brown and colleagues [32] provided 25 patients a 10 week program consisting of group classes by medicine, nursing or graduate studies students where each session was co-led by two to four students, Cusumano and colleagues’ [33] 12- week program led by physician assistant students consisted of an individualised one to one intervention of weekly goals, 12 individual meetings, six cooking classes and one supermarket tour provided to 28 patients. Across both studies, a mean weight loss between 2 to 3 kg [32, 33] and statistically significant reduction in mean BMI score 41.21 ± 10.64 kg/m^2^ to 40.13 ± 10.98 kg/m^2^ (*p* < 0.001) was achieved [33]. Whilst changes to BMI were statistically significant, these changes were not clinically meaningful [27]. A mean reduction of only 2–4% of total body weight was achieved through student-led interventions that provide an intensive group or individualised programs [32, 33].

### Blood pressure

Similarly, a statistically significant reduction in systolic blood pressure of 9.5 mmHg (CI 7.4, 11.5) and diastolic blood pressure of 5.7 mmHg (CI 4.4, 7.0) (*P* < 0.0001) amongst 496 patients with hypertension was observed after a free medical student-led clinic supervised by a physician [42]. There was no set contact schedule for patients accessing this service, as they were free to attend the clinic as required [42]. When medical appointment/s were provided to patients with diabetes through a student clinic, a statistically and clinically significant improvement in mean systolic blood pressure − 5.2 mmHg (*p* < 0.05) and diastolic blood pressure − 6.8 mmHg (*p* < 0.0001) was observed [43].

Whilst pairs of pharmacy students providing level two and three medication reviews observed non-statistically significant post-intervention systolic blood pressure (132.26 ± 12.9 mmHg) in 67 patients compared to the control group (127.98 ± 98 mmHg, *p* = 0.06, *n* = 66) and diastolic blood pressure (73.38 ± 6.8 mmHg compared to 70.97 ± 9.5 mmHg in the control group, *p* = 0.11) [31]. The change in systolic (132.48 ± 11.98 mmHg to 132.26 ± 12.9 mmHg) and diastolic blood pressure (73.22 ± 8.15 mmHg to 73.38 ± 6.8 mmHg) in the intervention group were not clinically meaningful [31]. A 12-month education program for 238 patients with diabetes provided by medical, pharmacy and physician assistant students a part of placement led to no change in systolic (136 mmHg to 136.9 mmHg, *p* = 0.217) or diastolic (81.3 mmHg to 82 mmHg, *p* = 0.073) blood pressure [39].

### Readmission rate

Only one study measured 30-day hospital readmission rate amongst heart failure patients as a post-intervention follow up outcome. Patients were counselled on medication management and lifestyle considerations such as weight, smoking, salt intake, alcohol and exercise at discharge and additionally provided a follow up phone call in three to 5 days post discharge [30]. Although one 52 min pharmacy student-led discharge counselling did elicit better understanding of medications in 89% of patients, no statistically significant changes in readmission rates compared to standard counselling by a nurse were observed [30]. Eleven percent were readmitted in the intervention group versus 9% readmitted in the control group (*p* = 0.80) within 30 days of discharge, and 11.1% of the intervention group were readmitted for heart failure [30].

### Student vs. professional

Two studies compared the student-led health intervention to a professional-led health intervention. Care provided by nursing or pharmacy students in a 30 min appointment in regards to self-management of diabetes was consistent with care provided by medical residents alone when observing mean HbA1c levels, LDL and blood pressure of both groups [35] (Table 2). This clinic serviced 384 patients with diabetes in a university clinic [35].

**Table 2:** Usual care provided by medical residents versus care provided by an interprofessional team of students [35]

	Medical residents		Interprofessional team of students		Between group
	Baseline (Mean ± SD)	Outcome (Mean ± SD)	Baseline (Mean ± SD)	Outcome (Mean ± SD)	*P*-value
HbA1c (%)	7.6 ± 1.7	7.5 ± 1.7	7.7 ± 1.7	7.7 ± 1.6	0.24
SBP (mmHg)	130 ± 20.5	130 ± 21.1	134 ± 21.0	134 ± 20.3	0.07
DBP (mmHg)	72.1 ± 12.0	71.8 ± 11.5	71.4 ± 10.6	71.0 ± 11.7	0.52
LDL (mg/dL)	107 ± 34.3	98.4 ± 31.9	106 ± 34.3	100 ± 31.3	0.64

When a 10-week group intervention for overweight and obese patients was led by an interprofessional (medicine, nursing, graduate studies and health professions) group of students, change in weight (*p* = 0.32) and BMI (− 1 kg/m^2^ vs. -0.7 kg/m^2^) was comparable to a 10-week group intervention taught by an interprofessional group consisting of a dietitian, psychologist and exercise physiologist [32]. Regardless of whether the intervention was led by students or professionals, high individual participant attendance rate was associated with greater weight loss amongst the participants (*p* < 0.001) [32].

## Discussion

Collectively, from the cardiovascular disease and risk factors sampled, student-led health interventions generally resulted in positive patient outcomes. While these outcomes do not necessarily pertain to the achievement of optimal clinical targets, it is important to note that even small improvements in the cardiovascular profile are considered to be clinically meaningful and may lead to a significant reduction in cardiovascular morbidity and mortality. The findings, therefore, provided moderate evidence [22] that student-led interventions improved cardiovascular markers and were comparable to professional-led interventions. Positive patient outcomes did not appear to strongly favour individual counselling interventions over appointment style interventions or group interventions and vice versa.

This review provides evidence from quasi-experimental studies that lacked randomisation and a control group. Nevertheless most studies, particularly studies amongst patients with diabetes, did reduce bias through their study design by measuring multiple outcome measures; completing and reporting follow up; measuring outcomes with a reliable method and using statistically appropriate methods (for critical appraisal of each study, see ‘Critical Appraisal of Randomised Controlled Trials’ and ‘Critical appraisal of quasi-experimental studies’ in Additional file 1).

The measurement of some patient outcomes was subject to a high risk of bias either due to only measuring one outcome measure or through a lack of detailed reporting of how the outcome was measured. Rojas and colleagues used LDL as the only patient outcome to determine hyperlipaemia control [41]. The determination of hyperlipaemia control could have been strengthened through reporting and considering a complete lipid study profile. This enables hyperlipaemia control to be determined by multiple measures which increases the reliability of the results. Although, medical student appointments were associated with statistically significant increases in BMI amongst patients with diabetes, body weight was not reported to support whether this negative result is clinically meaningful or not [39, 44]. Two retrospective studies did not report how weight was measured (i.e. with calibrated scales or patient reported weight) [32, 33]. One retrospective study did not report the method of measuring blood pressure (i.e. measured once or three times where the average of the second and third measures are used) [42]. This detail may impact the validity of the results.

Student-led interventions compared to professional-led interventions demonstrated comparable reductions in HbA1c, LDL, BMI and blood pressure [32, 35]. Along with the evidence that the quality of care from student-run clinics in USA are comparable to the national standards [47, 48], this finding supports emerging evidence that students can provide comparable care, when the environment to provide and lead care, exists. As this literature reduces the uncertainty of whether student-led interventions deliver the same level of care and intervention efficacy as professional-led services, the feasibility of student-led services as alternate service delivery model increases.

Although this review provides some evidence that student-led interventions can improve cardiovascular markers amongst patients at risk of cardiovascular disease, the findings were predominantly based on studies from the USA. This reduces the generalisability of these findings to other population groups and countries with alternate national health care delivery models. Additionally, the largest limitation of the included studies to enable replication, is the lack of intervention detail according to the TIDieR criteria (see ‘Patient intervention detail reported according to the TIDieR criteria’ and ‘Student intervention detail reported according to the TIDieR criteria’ in Additional file 1). Particularly amongst the student-led aspects of the interventions, details to describe the provider, procedures, schedule and intensity of the intervention are more scarce than the patient intervention [49]. Amongst the patient and student interventions, changes to the study design and intervention fidelity were commonly not reported. Whilst amongst the student intervention, what the intervention was, who provided it, how it was provided, where it was provided and how often or how much intervention, where also more scarcely reported than the patient intervention.

The very little detailed information within these studies to describe what the students did and therefore how the intervention impacted on their learning was unclear. A lack of description about what the experience is for students such as how much training, preparation and supervision is required, also limited the understanding of how much and what resources students require to achieve these patient outcomes and what the students’ learning outcomes are. To enhance implementation it will be important to understand the resources required including supervision and learning materials or frameworks in countries with different health systems to allow for determining the cost effectiveness of student-led interventions.

Additionally, this lack of reporting on what the students did sometimes impacted on how the patient outcomes could be interpreted. For example, whether medical students counselled patients, dispensed medication or provided both services were not reported [39, 44]. Without this detail, it was difficult to determine if the medical intervention could have resulted in weight loss and reduction in BMI as the reported patient outcomes.

Whilst the included studies lacked detailed reporting on the student intervention, there is literature that independently describes the training, supervision and resources required without clinical patient outcomes. For example, Froberg and colleagues detailed the administration, the organisation and the physical environment that students were exposed to when exploring student, patients and supervisor perceptions of participating in a student-run clinic [14]. Nevertheless, one of the included studies [32], recognised this limitation and likewise recommended that future research should investigate and report both patient and student outcomes. As this is a dual-intervention amongst patients and students, reporting the dual-intervention and outcomes will aid to inform the delivery and design of future services. As the prevalence of this alternate service delivery model is likely to increase with the increasing demands on healthcare services, this knowledge will assist in the feasibility of applying these services not only for underserved and socially disadvantaged populations alone but more broadly to usual healthcare provision.

## Conclusions

Student-led health interventions appear to improve cardiovascular markers amongst patients with cardiovascular disease and associated risk factors. Individualised student-led health interventions by medical and pharmacy students, amongst patients with diabetes, led to clinically and statistically significant improvements in HbA1c. Individualised and group-based programs of 10 or 12 weeks were associated with statistically significant weight loss amongst overweight and obese patients. Patient outcomes amongst student-led interventions were comparable to professional-led interventions. Additionally, this review illustrates the need for student-led health interventions to be thoroughly reported from both the patient and student perspectives and applied within a rigorous study design, to enable a meta-analysis of patient health outcomes from student-led health interventions and enable the intervention to be replicated. This knowledge will assist in determining the feasibility of student-led health interventions as an alternate service model to current provisions.

## Supplementary information

**Additional file 1.**

## Data Availability

Not applicable.

## Abbreviations

BP: Blood pressure
BMI: Body mass index
Euroqol VAS: Euroqol visual analogue scale
HbA1c: Haemoglobin -A1c
HDL: High density lipoprotein
LDL: Low density lipoprotein
TC: Total cholesterol
TIDieR: Template for intervention description and replication
