# Supplementary material for: Effect of student-led health interventions on patient outcomes for those with cardiovascular disease or cardiovascular disease risk factors: a systematic review
Source: BMC Cardiovasc Disord. 2020 Jul 11;20:332. doi: 10.1186/s12872-020-01602-1 (PMC7353670; doi:10.1186/s12872-020-01602-1)
Supplement: Supplementary file 1 — Additional file 1. [file 12872_2020_1602_MOESM1_ESM.docx]

**Supplementary Materials**

Search Strategies

| **Database searched** | **Search Strategy** | **Results**  **19/08/19** |
| --- | --- | --- |
| CINAHL | \| # \| Query \| Limiters/ Expander \| Results Results \| \| --- \| --- \| --- \| --- \| \| S1 \| TX (student* N4 (led OR run OR managed OR facilitated OR directed) ) AND (clinic OR clinics OR service* OR consult* OR care OR healthcare OR program* OR practice* OR model* OR initiative* OR intervention* OR promotion* OR centre* OR center*) \| Search modes - Boolean/ Phrase \| 1740 \|  \| \| S2 \| (MH "Obesity") OR (MH "Obesity, Morbid") \| Search modes - Boolean/ Phrase \| 75,589 \| \| S3 \| (MH "Overweight") \| Search modes - Boolean/ Phrase \| 0 \| \| S4 \| (MH "Diabetes Mellitus+") \| Search modes - Boolean/ Phrase \| 142,725 \| \| S5 \| (MH "Hypertension") \| Search modes - Boolean/ Phrase \| 48,465 \| \| S6 \| (MH "Blood Pressure") \| Search modes - Boolean/ Phrase \| 34,844 \| \| S7 \| (MH "Cardiovascular Diseases") OR (MH "Heart Diseases+") OR (MH "Vascular Diseases+") \| Search modes - Boolean/ Phrase \| 498,753 \| \| S8 \| (MH "Hyperlipidemia") OR (MH "Hypercholesterolemia") \| Search modes - Boolean/ Phrase \| 16,946 \| \| S9 \| TX (obes* or overweight or hyperten* or "blood pressure" or "cardiovascular disease" or "heart disease" or "vascular disease" or hyperlipid*mia or hypercholesterolemia) \| Search modes - Boolean/ Phrase \| 332,438 \| \| S10 \| S2 OR S3 OR S4 OR S5 OR S6 OR S7 OR S8 ORS9 \| Search modes - Boolean/ Phrase \| 781,453 \| \| S11 \| S1 AND S10 \| Search modes - Boolean/ Phrase \| 83 \| \| S12 \| S1 AND A10 \| Search modes – Boolean/ Phrase  Limiters- Age groups: Adolescents:13-18 years, Adult:19-44years, Middle Aged:45-64 years, Aged:65+, Language: English \| 32 \| | 32 |
| Medline | \| # \| Searches \|  \| \| --- \| --- \| --- \| \| 1 \| ((student* adj4 (led or run or managed or facilitated or directed)) and (clinic or clinics or service* or consult* or care or healthcare or program* or practice* or model* or initiative* or intervention* or promotion* or centre* or center*)).mp. \| 1747 \| \| 2 \| Obesity/ or Obesity, Morbid/ \| 187953 \| \| 3 \| Overweight/ \| 22769 \| \| 4 \| exp Diabetes Mellitus/ \| 406163 \| \| 5 \| Hypertension/ \| 227384 \| \| 6 \| Blood Pressure/ \| 268567 \| \| 7 \| cardiovascular diseases/ or exp heart diseases/ or exp vascular diseases/ \| 2285047 \| \| 8 \| exp Hyperlipidemias/ \| 64463 \| \| 9 \| (obes* or overweight or hyperten* or "blood pressure" or "cardiovascular disease" or "heart disease" or "vascular disease" or hyperlipid*mia or hypercholesterolemia) \| 1284202 \| \| 10 \| 2 OR 3 OR 4 OR 5 OR 6 OR 7 OR 8 OR 9 \| 3257557 \| \| 11 \| 1 AND 10 \| 87 \| \| 12 \| Limit 11 to (English language and humans and ("adolescent (13 to 18 years)" or "young adult (19 to 24 years)" or "adult (19 to 44 years)" or "young adult and adult (19-24 and 19-44)" or "middle age (45 to 64 years)" or "middle aged (45 plus years)" or "all aged (65 and over)" or "aged (80 and over)")) \| 30 \| | 30 |
| ProQuest Health & Medicine | **student* NEAR/4 (led OR run OR managed OR facilitated OR directed)**  **AND**  **clinic OR clinics OR service* OR consult* OR care OR healthcare OR program* OR practice* OR model* OR initiative* OR intervention* OR promotion* OR centre* OR center***  **AND**  **obes* OR "morbid* obes*" OR overweight OR diabet* OR hypertensi* OR "blood pressure" OR heart OR cardiac OR cardiovascular OR coronary OR vascular OR stroke OR "cerebrovascular accident*" OR arrhythmi* OR atrial OR myocardial OR hyperlipidemia OR cholesterol OR Hypercholesterolemia OR hypertriglyceridemia***  **Limit to: Full text**  **Additional limits: Age group: Adolescents 13-18years) Adults(19-44years), Middle aged (45-64 years), Aged (65+years), aged (80+years), Article, English** | 529 |
| PsycINFO | \| # \| Searches \|  \| \| --- \| --- \| --- \| \| 1 \| ((student* adj4 (led or run or managed or facilitated or directed)) and (clinic or clinics or service* or consult* or care or healthcare or program* or practice* or model* or initiative* or intervention* or promotion* or centre* or center*)).mp. \| 1973 \| \| 2 \| Obesity/ or Obesity, Morbid/ \| 23276 \| \| 3 \| Exp Overweight \| 244569 \| \| 4 \| exp DIABETES/ \| 16720 \| \| 5 \| HYPERTENSION/ \| 6486 \| \| 6 \| Blood Pressure/ \| 6885 \| \| 7 \| exp cardiovascular disorders/ \| 59669 \| \| 8 \| Cholesterol/ \| 2094 \| \| 9 \| (obes* or overweight or hyperten* or "blood pressure" or "cardiovascular disease" or "heart disease" or "vascular disease" or hyperlipid*mia or hypercholesterolemia).mp \| 91580 \| \| 10 \| 2 OR 3 OR 4 OR 5 OR 6 OR 7 OR 8 OR 9 \| 144850 \| \| 11 \| 1 AND 10 \| 21 \| \| 12 \| Limit 11 to (English language and adulthood<18+years>) \| 16 \| | 21 |
| Web of Science | (((student* NEAR/4 (led OR run OR managed OR facilitated OR directed)) AND (clinic OR clinics OR service* OR consult* OR care OR healthcare OR program* OR practice* OR model* OR initiative* OR intervention* OR promotion* OR centre* OR center*)) AND obes* OR “morbid* obes*” OR overweight OR diabet* OR hypertensi* OR "blood pressure" OR heart OR cardiac OR cardiovascular OR coronary OR vascular OR stroke OR "cerebrovascular accident*" OR arrhythmi* OR atrial OR myocardial OR hyperlipidemia OR cholesterol OR Hypercholesterolemia OR hypertriglyceridemia*)) Refined by: LANGUAGES: ( ENGLISH )  Timespan: All years. Indexes: SCI-EXPANDED, SSCI, A&HCI, CPCI-S, CPCI-SSH, ESCI, CCR-EXPANDED, IC. | 636 |
| Informit Health Database Collection | (obes* OR "morbid* obes*" OR overweight OR diabet* OR hypertensi* OR "blood pressure" OR heart OR cardiac OR cardiovascular OR coronary OR vascular OR stroke OR "cerebrovascular accident"* OR arrhythmi* OR atrial OR myocardial OR hyperlipidemia OR cholesterol OR Hypercholesterolemia OR hypertriglyceridemia*)  AND  ((clinic OR clinics OR service* OR consult* OR care OR healthcare OR program* OR practice* OR model* OR initiative* OR intervention* OR promotion* OR centre* OR center*))  AND  student (led OR run OR managed OR facilitated OR directed) | 4 |
| Cochrane Library | ((student* near/4 (led or run or managed or facilitated or directed)) and  (clinic or clinics or service* or consult* or care or healthcare or program* or practice* or model* or initiative* or intervention* or promotion* or centre* or center*)) and  (obes* or "morbid* obes*" or overweight or diabet* or hypertensi* or "blood pressure" or heart or cardiac or cardiovascular or coronary or vascular or stroke or "cerebrovascular accident*" or arrhythmi* or atrial or myocardial or hyperlipidemia or cholesterol or Hypercholesterolemia or hypertriglyceridemia* | 95 |
| Scopus | ( TITLE-ABS KEY ( ( ( ( student*  W/4  ( led  OR  run  OR  managed  OR  facilitated  OR  directed ) )  AND  ( clinic  OR  clinics  OR  service*  OR  consult*  OR  care  OR  healthcare  OR  program*  OR  practice*  OR  model*  OR  initiative*  OR  intervention*  OR  promotion*  OR  centre*  OR  center* ) ) ) ) )  AND  ( TITLE-ABS-KEY ( (  obes*  OR  "morbid* obes*"  OR  overweight*  OR  diabet*  OR  hypertensi*  OR  "blood pressure"  OR  heart  OR  cardiac  OR  cardiovascular  OR  coronary  OR  vascular  OR  stroke  OR  "cerebrovascular accident*"  OR  arrhythmi*  OR  atrial  OR  myocardial  OR  hyperlipidemia  OR  cholesterol  OR  hypercholesterolemia  OR  hypertriglyceridemia* ) )  AND  ( LIMIT-TO ( LANGUAGE ,  "English" ) ) | 208 |

Data Extraction Protocol

**Protocol for data extraction on Covidence:**

To extract all data available from the paper.

Watch <https://www.youtube.com/watch?v=iBqsKQDsW4k> re. editing data extraction forms to ensure all information is added.

Best to copy and paste information from the paper to help with consensus stage

Use notes to let other reviewer know if you have contacted author

Complete all fields

***Examples of extra Fields to add if not already present in the form:***

Identification:

- Year of publication
- Year that study occurred

Methods

- Description of intervention
- Duration of intervention
- Patient’s recruitment methods
- Strengths to data collection
- Limitations to data collection

Population

- Number of patient participants (e.g. 40)
- Number of patients included in data analysis (e.g. 12)
- Number of drop out and rate (e.g. 13, 20%)
- How was sample size calculated (if applicable)

Baseline characteristics

- All descriptive characteristics e.g. mean age, gender
- E.g. baseline HbA1c, other clinical markers

Interventions

- Describe intervention by adding the following characteristics as appropriate:
  – type of care provided (e.g. x reviews)
  - who the care was provided by (e.g. student nurse and doctor or student alone)
  - Tailoring (individualised, group or both)
  - length of the intervention – to describe number of session, schedule, duration and dose (e.g. 1 x 30 mins over 3months etc)
  - mode of delivery (e.g. face-to-face or internet or telephone or combination)
  - Location of the intervention (e.g. clinic, home or list all locations used)
  - What was provided? -include materials and procedures conducted
  - Intervention adherence or fidelity - (e.g. if assessed, provide a description of how Studendescribe the extent to which the intervention was delivered as planned)

**The fields above are from the TiDier Checklist to describe interventions**

- Describe student involvement if described by adding the following characteristics as appropriate :
  - Student involvement as volunteer or part of course work
  - Student’s discipline
  - Year level
  - Level of involvement (how many sessions involved)
  - Description of involvement
  - Relationship to coursework if relevant
  - Mutli-D or single discipline
  - Feedback provided or not

**Outcomes of interest are:**

1. Clinical outcomes associated with disease progression: e.g. lipid studies, HbA1c, BP, adverse event
2. Student outcomes

Add outcomes based on study

**Please check reference lists for other relevant articles**

**Results Summary Table**

| **Author, year** | **Population** | **Patient Intervention** | **Results** |
| --- | --- | --- | --- |
| Adams, 2015 | Type 2 Diabetes  Intervention (n=67)  Control (n=56) | Intervention:  Two student led level 3 medication review provided by pharmacy student under supervision  Control: standard care | \|  \| Intervention \| \| Control \| \|  \| \| --- \| --- \| --- \| --- \| --- \| --- \| \|  \| Mean \| SD \| Mean \| SD \| P-value \| \| Baseline HbA1c mmol/mol \| 56.81 \| 11.12 \| 59.71 \| 13.92 \|  \| \| Outcome HbA1c mmol/mol \| 56.32 \| 11.5 \| 59.68 \| 13.2 \| 0.14 \| \| Baseline TC mmol/L \| 4.14 \| 0.99 \| 4.19 \| 0.91 \|  \| \| Outcome TC mmol/L \| 4.22 \| 1.0 \| 4.01 \| 0.8 \| 0.47 \| \| Baseline SBP mmHg \| 132.48 \| 11.98 \| 131.65 \| 10.9 \|  \| \| Outcome SBP mmHg \| 132.36 \| 12.9 \| 127.98 \| 11.9 \| 0.06 \| \| Baseline DBP mmHg \| 73.22 \| 8.15 \| 72.13 \| 9.54 \|  \| \| Outcome DBP mmHg \| 73.38 \| 6.8 \| 70.97 \| 9.5 \| 0.11 \| |
| Brown, 2015 | Low income overweight and obese community residents  N =25 | 10 group community classes co-taught by between 2 to 4 students weekly over 10 weeks. | \|  \| Intervention \| \| \| --- \| --- \| --- \| \|  \| Mean \| SD \| \| Baseline weight (kg) \| 112.99 \| 36.84 \| \| Outcome weight (kg) \| 111.03 \| 36.91 \| \| Baseline BMI (kg/m^2^) \| 41.43 \| 11.85 \| \| Outcome BMI (kg/m^2^) \| 40.72 \| 11.89 \| \| Percent weight change \| -2.51 \| 2.08 \| |
| Cusumano, 2017 | Overweight or obese patients based on BMI score.  N=28 | 12 individual meetings to set goals and strategies, 6 cooking classes and 1 supermarket tour providing 19 sessions over 12 weeks. | \|  \| Intervention \| \|  \| \| --- \| --- \| --- \| --- \| \|  \| Mean \| SD \| P-value \| \| Baseline weight (kg) \| 111.98 \| 33.47 \|  \| \| Outcome weight (kg) \| 109.00 \| 33.93 \| <0.001 \| \| Baseline BMI (kg/m^2^) \| 41.21 \| 10.64 \|  \| \| Outcome BMI (kg/m^2^) \| 40.13 \| 10.98 \| <0.001 \| |
| Gorrindo, 2014 | Type 2 Diabetes  N= 43 | Visits and phone calls regarding clinical care provided by PHE students to provide follow up every one to two moths or as needed over 12 months. | \|  \| Intervention \| \| P-value \| \| --- \| --- \| --- \| --- \| \|  \| Mean \| SD \|  \| \| Outcome HbA1c \| 9.6 \| NP \| <0.0001 \| |
| Janson, 2009 | Type 2 Diabetes  Intervention (n= 384)  Control (n=163) | Individual 30-minute appointment and follow up for individual self-management activities by nursing practitioner or pharmacy students. | \|  \| Intervention \| \| Control \| \|  \| \| --- \| --- \| --- \| --- \| --- \| --- \| \|  \| Mean \| SD \| Mean \| SD \| P-value \| \| Baseline HbA1c \| 7.7 \| 1.7 \| 7.6 \| 1.7 \| 0.60 \| \| Outcome HbA1c \| 7.7 \| 1.6 \| 7.5 \| 1.7 \| 0.24 \| \| Baseline LDL \| 106 \| 34.3 \| 107 \| 36.3 \| 0.90 \| \| Outcome LDL \| 100 \| 31.3 \| 98.4 \| 31.9 \| 0.64 \| \| Baseline SBP \| 134 \| 21 \| 130 \| 20.5 \| 0.09 \| \| Outcome SBP \| 134 \| 20.3 \| 130 \| 21.1 \| 0.07 \| \| Baseline DBP \| 71.4 \| 10.6 \| 72.1 \| 12 \| 0.54 \| \| Outcome DBP \| 71 \| 11.7 \| 71.8 \| 11.5 \| 0.52 \| |
| Kahkoska, 2018 | Type 2 diabetes  N=29 | Team of trans-disciplinary trainees (medical, nursing, pharmacy students) working together to perform triage, medication reconciliation, brief history and physical exam followed by one 60 to 90 min shared medical appointment led by patients. | \|  \| Intervention \| \| \| --- \| --- \| --- \| \|  \| Mean \| SD \| \| Baseline HbA1c \| 9.7 \| 1.6 \| \| Outcome HbA1c \| 9.2 \| 1.4 \| |
| Lee, 2016 | Type 2 Diabetes  N=22 | Six classes and 12 weekly phone calls over 12 weeks delivered by medical students and resident physicians. | \|  \| Intervention \| \| \| --- \| --- \| --- \| \|  \| % \| Range \| \| Baseline HbA1c \| 9.2 \| 6.0-12.5 \| \| Outcome HbA1c \| 8.0 \| 5.9-11.4 \| |
| Martin, 2015 | Type 2 Diabetes  N=48 | One off clinic visits provided by pharmacy students | \|  \| Uncontrolled diabetes \| \| Controlled diabetes \| \| \| --- \| --- \| --- \| --- \| --- \| \|  \| Median \| % \| Median \| % \| \| Baseline HbA1c \| 78.1 \| 9.3 \| 44.3 \| 6.2 \| \| Outcome HbA1c \| 59.8 \| 7.6 \| 46.4 \| 6.4 \| \| P-value \| Not significant \| \| 0.004 \| \| |
| Mehta, 2016 | Type 1 or Type 2 Diabetes  N=68 | Individual education conducted by medical students about advantage of primary care in the management of diabetes and preventing long-term complications over 9 months | \|  \| Attended 2 or more appointments \| \| Attended less than 2 appointments \| \| \| --- \| --- \| --- \| --- \| --- \| \|  \| Mean \| SD \| Mean \| SD \| \| Baseline HbA1c \| 9.5 \| 2.3 \| 8.9 \| 3.1 \| \| Outcome HbA1c \| 8.3 \| 2.2 \| 8.3 \| 2.1 \| \| P-value \| 0.008 \| \| 0.4 \| \| |
| Nagelkerk, 2018 | Diabetes  N=250 | Inter-professional collaborative practice education program provided by an inter-professional team of students (medical, pharmacy and physician assistant students) with the practice team. | \|  \| Intervention \| \| \| \| --- \| --- \| --- \| --- \| \|  \| Mean \| P-value \| \| Baseline HbA1c \| 7.3 \|  \| \| Outcome HbA1c \| 7.2 \| 0.346 \| \| Baseline SBP \| 136 \|  \| \| Outcome SBP \| 136.9 \| 0.217 \| \| Baseline DBP \| 81.3 \|  \| \| Outcome DBP \| 82.0 \| 0.073 \| \| Baseline HDL \| 49.7 \|  \| \| Outcome HDL \| 50.8 \| 0.126 \| \| Baseline LDL \| 102.3 \|  \| \| Outcome LDL \| 97.6 \| 0.171 \| \| Baseline glucose \| 156.7 \|  \| \| Outcome glucose \| 148.7 \| 0.110 \| |
| Nuffer, 2012 | Type 1 or Type 2 Diabetes | Six one hour, one-to-one diabetes self-management sessions by pharmacy students over 6 months. | \|  \| Intervention \| \| P-value \| \| --- \| --- \| --- \| --- \| \|  \| Mean \| SD \|  \| \| Baseline HbA1c \| 7.7 \| 2.0 \|  \| \| 6mo post intervention HbA1c \| 6.8 \| 1.1 \| <0.001 \| \| Baseline TC \| 181.3 \| 45.7 \|  \| \| 6mo post intervention TC \| 167.2 \| 39.2 \| <0.001 \| \| Baseline SBP \| 132.3 \| 16.8 \|  \| \| 6mo post intervention SBP \| 128 \| 14.6 \| <0.001 \| \| Baseline DBP \| 77.8 \| 10.9 \|  \| \| 6mo post intervention DBP \| 75.3 \| 9.8 \| <0.001 \| \| Baseline LDL \| 104.1 \| 42 \|  \| \| 6mo post intervention LDL \| 92.1 \| 33.4 \| <0.001 \| \| Baseline HDL \| 41.7 \| 12.2 \|  \| \| 6mo post intervention HDL \| 42.9 \| 14.3 \| 0.15 \| \| Baseline TG \| 192.2 \| 116 \|  \| \| 6mo post intervention TG \| 161 \| 91.2 \| <0.001 \| |
| Rojas, 2015 | Hyperlipidemia  N= 96 | Free appointment with medical students and dispensing of medications free of change. Follow up between 6 weeks and 18 months after initial appointment. | \|  \| Intervention \| \| P-value \| \| --- \| --- \| --- \| --- \| \|  \| Mean \| SD \|  \| \| Baseline LDL mg/dl \| 135.8 \| 37.2 \|  \| \| Follow up LDL mg/dl \| 101.3 \| 34.6 \| <0.001 \| |
| Smith, 2014 | Diabetes  N=182 | Individualized clinic sessions by medical students. | \|  \| Intervention \| \| P-value \| \| --- \| --- \| --- \| --- \| \|  \| Mean \| SD \|  \| \| Baseline HbA1c \| 9.15 \| 2.5 \|  \| \| 6mo post intervention HbA1c \| 8.19 \| 2.15 \| 0.001 \| \| Baseline LDL \| 116.34 \| 43.6 \|  \| \| 6mo post intervention LDL \| 87.21 \| 32.19 \| 0.001 \| \| Baseline HDL \| 46.11 \| 13.84 \|  \| \| 6mo post intervention HDL \| 49.35 \| 13.00 \| 0.001 \| \| Baseline TG \| 230.18 \| 191.87 \|  \| \| 6mo post intervention TG \| 159.64 \| 84.28 \| 0.001 \| \| Baseline SBP \| 131.89 \| 18.2 \|  \| \| 6mo post intervention SBP \| 126.65 \| 18.77 \| 0.05 \| \| Baseline DBP \| 81.86 \| 12.08 \|  \| \| 6mo post intervention DBP \| 75.08 \| 11.79 \| 0.001 \| |
| Smith, 2017 | Hypertension  N=496 | Free appointment by medical students under physician supervision. Follow up visit 9 to 15 months later. | \|  \| Intervention \| \| P-value \| \| --- \| --- \| --- \| --- \| \|  \| Mean \| SD \|  \| \| Baseline SBP \| 141.6 \| 21.8 \|  \| \| Outcome SBP \| 132.1 \| 17.3 \| <0.001 \| \| Baseline DBP \| 85.1 \| 13.2 \|  \| \| Outcome DBP \| 79.4 \| 10.8 \| <0.001 \| |
| Stroup, 2003 | Diabetes  Intervention (n=30)  Control (n=40) | Home visitation or phone call program provided by pharmacy students over 2 years. | \|  \| Intervention \| \| Control \| \| P-value \| \| --- \| --- \| --- \| --- \| --- \| --- \| \|  \| Mean \| SD \| Mean \| SD \|  \| \| Baseline HbA1c \| 11.2 \| 1.3 \| 10.7 \| 1.6 \|  \| \| Outcome HbA1c \| 10.0 \| 2.0 \| 9.9 \| 2.5 \|  \| \| Change \| -1.2 \|  \| -0.8 \|  \| 0.4678 \| \| Baseline TC \| 203 \| 43.4 \| 193.2 \| 30.1 \|  \| \| Outcome TC \| 195.6 \| 46.3 \| 184.5 \| 39.4 \|  \| \| Change \| -8.5 \|  \| -5.0 \|  \| 0.8047 \| \| Baseline SBP \| 130.8 \| 14.5 \| 121.3 \| 15.3 \|  \| \| Outcome SBP \| 130.8 \| 15.4 \| 125 \| 15 \|  \| \| Change \| -0.9 \|  \| +3.0 \|  \| 0.4545 \| \| Baseline DBP \| 79.1 \| 7.2 \| 74.4 \| 9.1 \|  \| \| Outcome DBP \| 76.9 \| 8.6 \| 74.1 \| 7.8 \|  \| \| Change \| -2.5 \|  \| -0.5 \|  \| 0.4955 \| |
| Szkiladz, 2013 | Heart failure  Intervention (n=86)  Control (n=94) | Additional discharge counseling by pharmacy students compared to no discharge counseling by pharmacy students on medication and lifestyle considerations. | \|  \| Intervention \| Control \| P-value \| \| --- \| --- \| --- \| --- \| \|  \| % \| % \|  \| \| Adjusted heart failure readmissions \| 10.5 \| 8.5 \| 0.8 \| \| Adjusted heart failure readmission \| 11.1 \| 8.1 \| 0.52 \| |

**Critical Appraisal of Randomised Controlled Trials**

| **Author, year** | **Adams, 2015** | **Stroup, 2003** |
| --- | --- | --- |
| **Chronic Disease** | T2DM | Diabetes |
| 1. Was true randomisation used for assignment of participants to treatment groups? | Y | Unclear |
| 2. Was allocation to treatment groups concealed? | Y | N |
| 3. Were treatment groups similar at the baseline? | Y | Y |
| 4. Were participants blind to treatment assignment? | N | N |
| 5. Were those delivering treatment blind to treatment assignment? | N | N |
| 6. Were outcomes assessors blind to treatment assignment? | Y | N |
| 7. Were treatment groups treated identically other than the intervention of interest? | Y | Unclear |
| 8. Was follow up complete and if not, were differences between groups in terms of their follow up adequately described and analysed? | Y | N |
| 9. Were participants analysed in the groups to which they were randomised? | Y | N |
| 10. Were outcomes measured in the same way for treatment groups? | Y | Y |
| 11. Were outcomes measured in a reliable way? | Y | Y |
| 12. Was appropriate statistical analysis used? | Y | Y |
| 13. Was the trial design appropriate, and any deviations from the standard RCT design (individual randomisation, parallel groups) accounted for in the conduct and analysis of the trial? | Y | Y |
| *Overall Appraisal (Include, Exclude, Seek further info)* | Include | Include |
| *Comments (Including reason for exclusion)* | No outcome timepoint data measured. Trial was unblinded. | Method of randomisation applied, allocation concealment and number of patients included in 2-year outcome data was not stated. Patients continued to see their physicians as scheduled. |

Critical appraisal of quasi-experimental studies

| **Author, year** | **Brown, 2015** | **Janson, 2009** | **Kahkoska, 2018** | **Lee, 2016** | **Nagelkerk, 2018** | **Szkiladz, 2013** | **Cusumano, 2017** | **Gorrindo, 2014** | **Martin, 2015** | **Mehta, 2016** | **Nuffer, 2012** | **Rojas, 2015** | **Smith, 2014** | **Smith, 2017** |
| --- | --- | --- | --- | --- | --- | --- | --- | --- | --- | --- | --- | --- | --- | --- |
|  |  |  |  |  |  |  |  |  |  |  |  |  |  |  |
|  |  |  |  |  |  |  |  |  |  |  |  |  |  |  |
| **Chronic Disease** | Overweight and Obestiy | Diabetes | Diabetes | Diabetes | Diabetes | Heart Failure | Overweight and Obestiy | Diabetes | Diabetes | Diabetes | Diabetes | Hyperlipidaemia | Diabetes | Hypertension |
| **1. Is it clear in the study what is the cause' and what is the 'effect' (i.e. there is no confusion about which variable comes first)?** | Y | Y | Y | Y | Y | Y | Y | Y | Y | Y | Y | Y | Y | Y |
| **2. Were the participants included in any comparisons similar?** | Y | NA | NA | NA | NA | Y | NA | NA | NA | NA | NA | NA | NA | NA |
| **3. Were the participants included in any comparisons receiving similar treatment/care, other than the exposure or intervention of interest?** | Y | NA | NA | NA | NA | Y | Unclear | NA | NA | NA | NA | NA | NA | NA |
| **4. Was there a control group?** | N | Y | N | N | N | Y | N | N | N | N | N | N | N | N |
| **5. Were there multiple measurements of the outcome both pre and post the intervention/ exposure?** | Y | Y | N | Y | Y | Y | Y | Y | Unclear | Y | Y | N | Y | Unclear |
| **6. Was follow up complete and if not, were differences between groups in terms of their follow up adequately described and analysed?** | N | Y | Y | NA | Unclear | Y | Y | Y | Y | Y | Y | Y | Y | Y |
| **7. Were the outcomes of participants included in any comparisons measured in the same way?** | Y | Y | Y | NA | Y | N | NA | NA | NA | NA | NA | Y | NA | NA |
| **8. Were outcomes measured in a reliable way?** | Unclear | Y | Y | Y | Y | Y | Unclear | Y | Y | Y | Y | Y | Y | Unclear |
| **9. Was appropriate statistical analysis used?** | Y | Y | Y | Y | Y | Y | Unclear | Y | Y | Y | Y | Y | Y | Y |
| *Overall Appraisal (Include, Exclude, Seek further info)* | Include | Include | Include | Include | Include | Include | Include | Include | Include | Include | Include | Include | Include | Include |
| *Comments (Including reason for exclusion)* | the |  | HbA1c was the only outcome measure. |  | Unclear why not all patients have lipid study results | Lacked detail on number of patients who did not receive follow up and readmitted. | Was a chart audit so described BMI and weight as measures collected from charts. Unclear how weight was originally measured. | Retrospective study. | Retrospective study. | Retrospective study. | Retrospective study. | Only collected LDL data as outcome measure to determine hypelipidaemia control. | Retrospective study. | Collect BP data from chart, unclear how original BP was measured. Only measured BP as outcome measure for hypertension control. |

Patient intervention detail reported according to the TIDieR criteria (42)

| Author, year | Patient intervention | | | | | | | |
| --- | --- | --- | --- | --- | --- | --- | --- | --- |
|  | What | Who provided | How | Where | When and how much | Tailoring | Changes | Fidelity |
| Adams et al, 2015 | Yes | Yes | Yes | Yes | Yes | Yes | No | No |
| Brown et al, 2015 | Yes | Yes | Yes | Yes | Yes | No | No | No |
| Cusumano et al, 2017 | Yes | Yes | Yes | Yes | Yes | Yes | No | No |
| Gorrindo et al, 2014 | Yes | Yes | Yes | Yes | No | Yes | No | No |
| Janson et al, 2009 | Yes | Yes | Yes | Yes | Yes | Yes | No | No |
| Kahkosa et al, 2018 | Yes | Yes | Yes | Yes | Yes | Yes | No | No |
| Lee et al, 2016 | Yes | Yes | Yes | Yes | Yes | No | No | No |
| Martin et al, 2015 | Yes | Yes | Yes | Yes | Yes | Yes | No | No |
| Mehta et al, 2016 | Yes | Yes | Yes | Yes | Yes | Yes | No | No |
| Nagelkerk et al, 2018 | Yes | Yes | Yes | Yes | No | No | No | No |
| Nuffer et al, 2012 | Yes | Yes | Yes | Yes | Yes | Yes | No | No |
| Rojas et al 2015 | Yes | Yes | Yes | Yes | No | Yes | No | No |
| Smith et al, 2014 | Yes | Yes | Yes | Yes | Yes | Yes | No | No |
| Smith et al, 2017 | Yes | Yes | Yes | Yes | No | Yes | No | No |
| Stroup et al, 2003 | Yes | Yes | Yes | Yes | No | Yes | No | No |
| Szkiladz et al, 2013 | Yes | Yes | Yes | Yes | Yes | Yes | No | No |

Student intervention detail reported according to the TIDieR criteria (42)

| Author, year | Student intervention | | | | | | | |
| --- | --- | --- | --- | --- | --- | --- | --- | --- |
|  | What | Who provided | How | Where | When and how much | Tailoring | Changes | Fidelity |
| Adams et al, 2015 | Yes | Yes | Yes | Yes | Yes | Yes | No | No |
| Brown et al, 2015 | Yes | Yes | Yes | No | Yes | Yes | No | No |
| Cusumano et al, 2017 | Yes | Yes | No | No | No | Yes | No | No |
| Gorrindo et al, 2014 | Yes | Yes | No | No | Yes | Yes | No | No |
| Janson et al, 2009 | Yes | Yes | Yes | No | Yes | Yes | No | No |
| Kahkosa et al, 2018 | Yes | No | Yes | Yes | No | No | No | No |
| Lee et al, 2016 | Yes | Yes | Yes | No | Yes | Yes | No | No |
| Martin et al, 2015 | Yes | No | Yes | Yes | No | Yes | No | No |
| Mehta et al, 2016 | Yes | Yes | No | No | No | No | No | No |
| Nagelkerk et al, 2018 | Yes | Yes | Yes | Yes | Yes | Yes | No | No |
| Nuffer et al, 2012 | Yes | Yes | Yes | Yes | Yes | Yes | No | No |
| Rojas et al 2015 | No | No | Yes | No | No | No | No | No |
| Smith et al, 2014 | No | No | Yes | No | No | No | No | No |
| Smith et al, 2017 | No | No | Yes | No | No | No | No | No |
| Stroup et al, 2003 | No | No | Yes | No | No | No | No | No |
| Szkiladz et al, 2013 | Yes | Yes | Yes | Yes | No | Yes | No | No |
